# Boerhaave syndrome complicated by ruptured esophageal varices: a case report

**DOI:** 10.1186/s44215-025-00226-1

**Published:** 2025-11-07

**Authors:** Kosei Uehara, Makoto Sohda, Kengo Kuriyama, Akiharu Kimura, Akihiko Sano, Makoto Sakai, Ken Shirabe, Hiroshi Saeki

**Affiliations:** 1 Department of General Surgical Science, Division of Gastroenterological Surgery, Gunma University Graduate School of Medicine, Maebashi, Gunma 371-8511 Japan; 2 Department of Gastroenterological Surgery, Gunma Prefectural Cancer Center, Ota, Gunma Japan; 3 Department of General Surgical Science, Gunma University Graduate School of Medicine, Maebashi, Gunma Japan

**Keywords:** Boerhaave syndrome, Esophageal perforation, Esophageal varices, Liver cirrhosis

## Abstract

**Background:**

Boerhaave syndrome is a life-threatening condition caused by a spontaneous full-thickness rupture of the esophagus, typically following forceful vomiting. Esophageal variceal rupture is another potentially fatal condition commonly observed in patients with liver cirrhosis. These two conditions are usually reported independently, and their coexistence is extremely rare. Managing this combination is particularly challenging because of the need for both infection control and hemostasis in the presence of portal hypertension.

**Case presentation:**

We report the case of a 51-year-old man with liver cirrhosis (Child–Pugh Grade B) and a history of endoscopic variceal ligation, who presented with hematemesis followed by vomiting. He had not consumed any alcohol at that time and exhibited no signs of melena. Computed tomography revealed mediastinal emphysema, right-sided hemopneumothorax, and esophageal perforation. Endoscopy confirmed a lower esophageal tear with active variceal bleeding. Emergency surgery was performed via a right thoracotomy. A 35-mm longitudinal tear in the lower esophagus was identified and sutured; however, owing to tissue fragility and signs of infection, a T-tube was placed at the perforation site. The patient had an uneventful postoperative course, resumed oral intake on postoperative day 17, and was discharged without any complications on day 46.

**Conclusions:**

This was a rare case of Boerhaave syndrome complicated by ruptured esophageal varices. Surgical management with T-tube drainage was effective in controlling both infection and bleeding. Patients with esophageal varices may be at an increased risk of esophageal rupture, especially following vomiting. Awareness of this association is essential for timely diagnosis and management.

## Background

Boerhaave syndrome is a life-threatening condition characterized by a full-thickness rupture of the esophageal wall due to a sudden increase in intraesophageal pressure, typically followed by a forceful vomiting. Prompt diagnosis and immediate treatment are crucial because any delay significantly increases the mortality risk [1–4].

Esophageal varices are thin-walled veins in the esophagus that are prone to massive bleeding if ruptured.

Boerhaave syndrome and esophageal variceal rupture are typically reported as separate clinical entities, and cases in which both conditions coexist are rare. Surgical intervention is often required for esophageal perforations, but perioperative management is particularly challenging in patients with concomitant esophageal varices and liver cirrhosis.

This report describes a unique case of Boerhaave syndrome and esophageal variceal rupture; however, prompt surgery proved to be lifesaving. An exhaustive search revealed no prior reports of this exceptionally rare co-occurrence of Boerhaave syndrome and ruptured esophageal varices.

## Case presentation

**Fig. 1:**
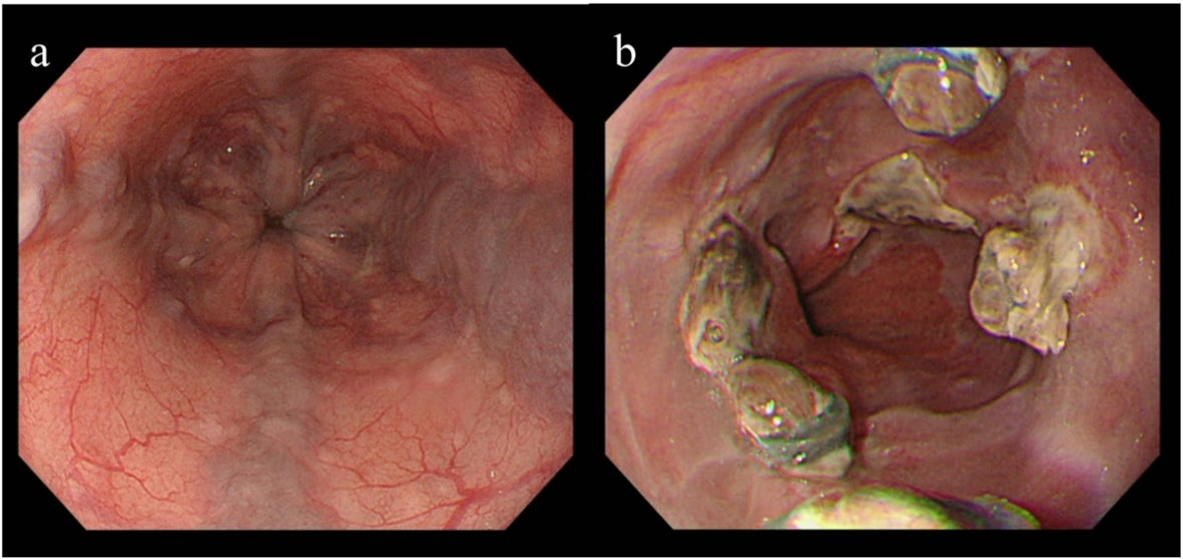
**a** Untreated esophageal varices were noted in the esophagus. **b** This endoscopic image was obtained during follow-up after endoscopic variceal ligation (EVL) for esophageal varices

A 51-year-old man had a history of alcoholic liver cirrhosis (Child–Pugh Grade B) and esophageal varices. He had a long-standing history of heavy alcohol use—previously consuming up to ten 500-mL beers per day (5% ABV; ≈200 g ethanol/day) and, in the period immediately prior to presentation, approximately three 500-mL beers daily (≈60 g ethanol/day). The patient had previously undergone endoscopic variceal ligation (EVL) at another hospital (Fig. 1).

The patient presented to his previous physician for emergency evaluation of vomiting and hematemesis. The patient did not consume alcohol at the time of vomiting and exhibited no symptoms such as melena. Approximately 1 h after lunch, he vomited food residue mixed with blood, followed by the expulsion of fresh blood.

Enhanced computed tomography (CT) revealed mediastinal emphysema and right hemopneumothorax. Based on the clinical course and imaging findings, Boerhaave syndrome was diagnosed. Owing to respiratory instability, a right chest tube was immediately inserted. The patient was transferred to our hospital for further management.

On arrival, the patient exhibited dyspnea with an oxygen saturation of 95% on a face mask, delivering 6 L of oxygen. Additionally, he presented with hemodynamic instability, as evidenced by a blood pressure of 100/78 mmHg and tachycardia (heart rate of 135 bpm). Thoracic drainage revealed a persistent bloody drainage. Arterial blood gas analysis revealed hyperlactatemia (6.1 mmol/L). Blood tests revealed hemoglobin 12.3 g/dL, total bilirubin 3.0 mg/dL, aspartate aminotransferase 141 U/I, alanine aminotransferase 45 U/I, gamma-glutamyl transferase 549 U/I, ammonia 113 μg/L, and prothrombin time ratio 1.58. Given the overall unstable condition, the patient was admitted to the intensive care unit and intubated for mechanical ventilation.

**Fig. 2:**
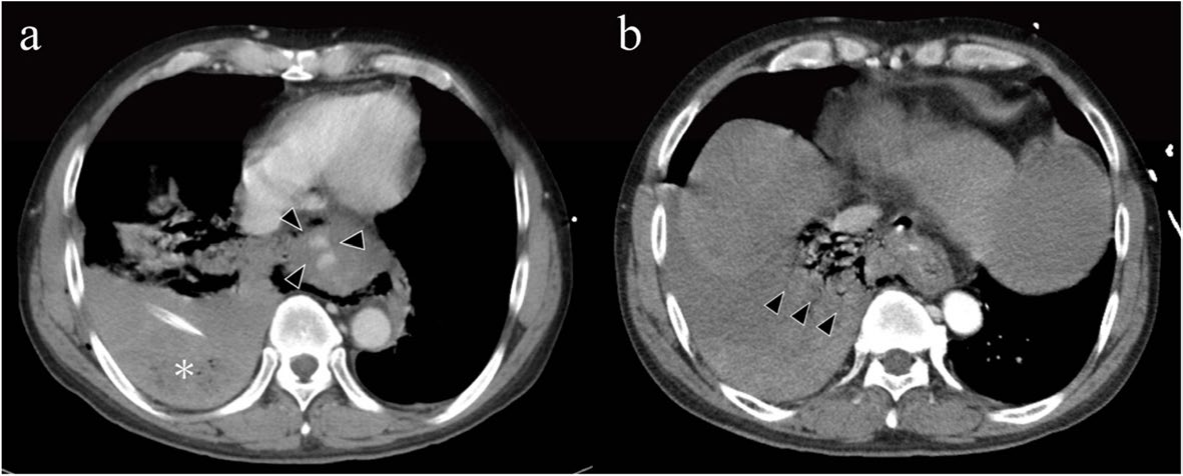
**a** Computed tomography shows pleural effusion in the right thoracic cavity (asterisk) and contrast leak into the esophagus (triangle). **b** Disruption of the esophageal wall in the right thoracic cavity (triangle)

CT scan revealed pleural effusion with air in the right thoracic cavity, contrast leakage into the esophagus (Fig. 2), and disruption of the lower esophageal wall.

**Fig. 3:**
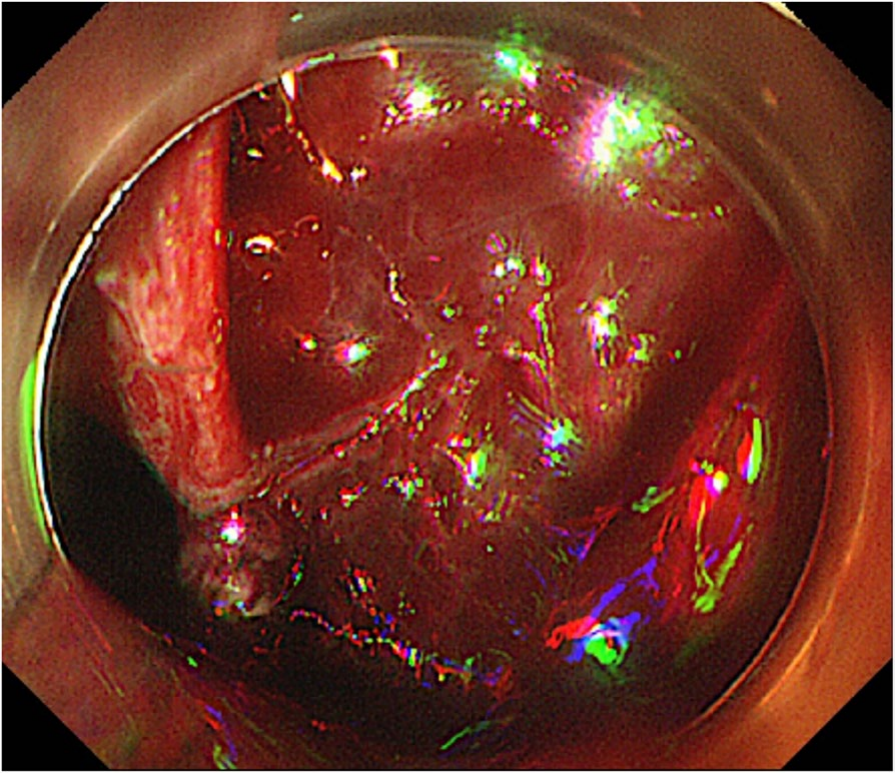
Endoscopy confirmed a laceration in the lower esophagus and bleeding from a ruptured varicocele at the same site

Additionally, varices and extrahepatic collateral vessels were observed in the esophagus, suggesting that Boerhaave syndrome is complicated by ruptured esophageal varices. Endoscopy confirmed a laceration in the lower esophagus and bleeding from a ruptured varicocele at the same site located at the 2 o’clock position (Fig. 3). Clipping failed to stop the bleeding.

**Fig. 4:**
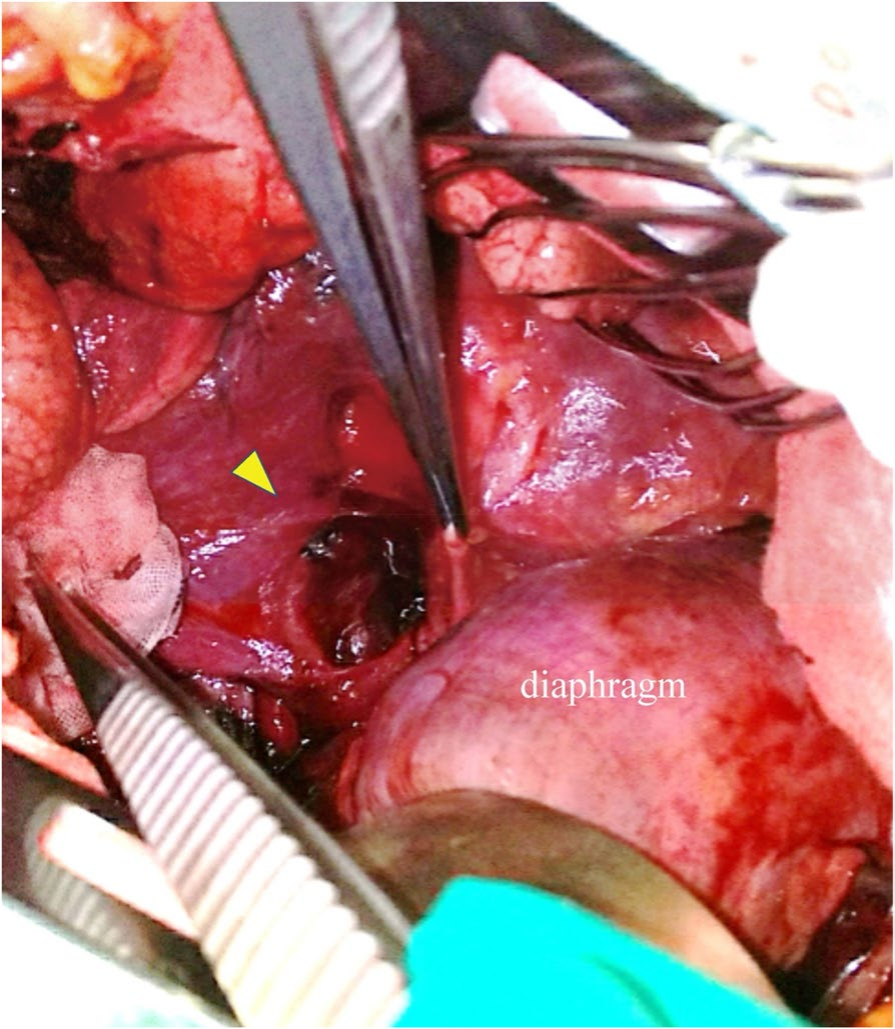
A perforation (triangle) was observed extending from the lower esophagus to the esophagogastric junction, involving the esophageal wall and continuing into the right thoracic cavity

We diagnosed Boerhaave syndrome with ruptured esophageal varices and opted for surgery to control the bleeding and potential infection. Under general anesthesia, right thoracotomy was performed through the seventh intercostal space. Approximately 3 L of blood with clots was found in the chest cavity. A 35-mm tear was identified in the lower right esophagus, which was sutured to stop the bleeding (Fig. 4). As no discrete bleeding point could be identified, hemostasis was achieved by placing broad single-layer sutures incorporating the pleura and the margins of the esophageal perforation.

**Fig. 5:**
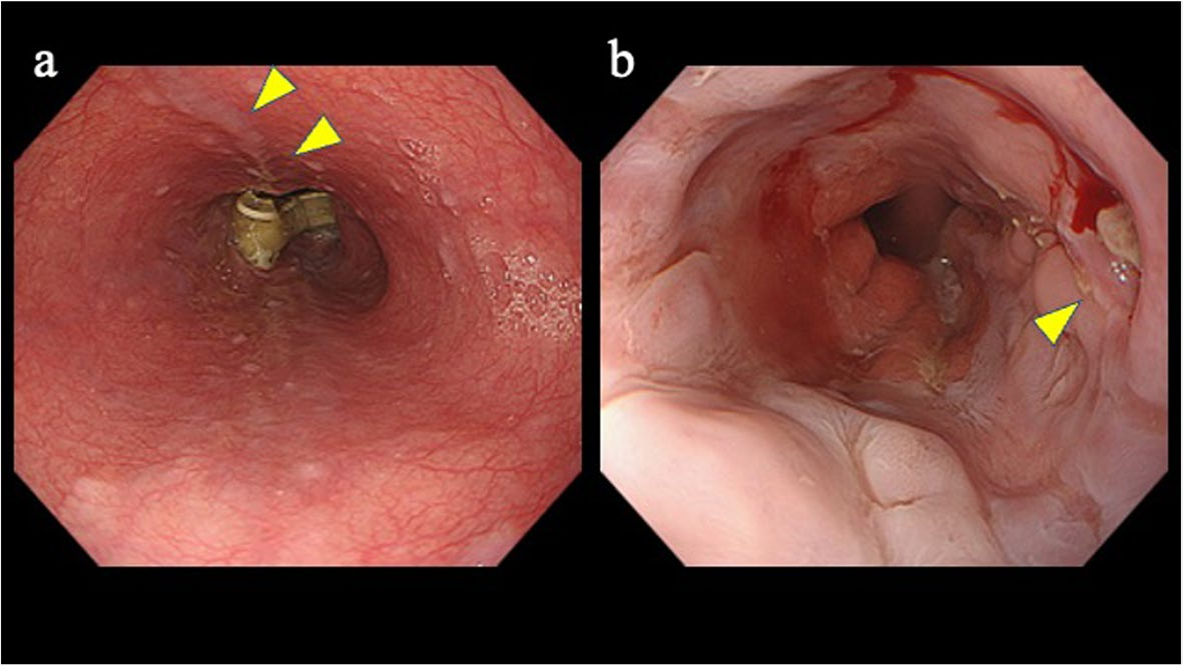
**a** A T-tube is in place. Esophageal varices are present along the anterior wall (triangle). **b** After endoscopy-guided T-tube removal, slight oozing from the removal site was noted, but the suture line appeared intact

Owing to signs of infection and a fragile esophageal wall, an 18-Fr T-tube drain was placed. The perforation site was covered with surrounding pleural and pericardial fat. A thoracic drain was placed in the upper thoracic cavity (24-Fr), and above the diaphragm (28-Fr). While mediastinal drainage was considered, percutaneous insertion was avoided because of the prominent subcutaneous varices. The patient’s postoperative course was uneventful. Until oral intake was initiated, total parenteral nutrition was administered via a central venous catheter. He started eating on postoperative day 17, and the T-tube was removed on day 37 without prior exchange, under endoscopic guidance. After T-tube removal, the patient was kept NPO for 4 days, and he was discharged home on day 46 with no complications (Fig. 5).

## Discussion and Conclusions

Boerhaave syndrome is a relatively rare but life-threatening condition that involves esophageal perforation due to a sudden increase in intraesophageal pressure caused by a closed pharynx [5]. Alcohol consumption and overeating are the common risk factors. Perforation most frequently occurs in the lower thoracic esophagus, with left-sided predominance [6].

Treatment delay is the most critical prognostic factor for esophageal perforation. The mortality rate is reported to be < 10% in patients who receive management within 24 h of onset, whereas it increases to approximately 30% in those treated after 24 h [1–4].

The treatment options for Boerhaave’s syndrome include surgery and conservative management. However, surgery is generally the preferred approach [1, 7].

Conservative treatment involves fasting, administration of proton pump inhibitors, and broad-spectrum antibiotics. This approach is considered if infection can be adequately controlled [8–10]. Endoscopic management may be considered in patients who are poor surgical candidates or in whom the perforation is small. For larger defects that are not amenable to endoscopic clipping, placement of a covered self-expanding esophageal stent has been reported to be useful [7, 11, 12]. Surgery focuses on controlling infection and closing the perforation site [13]. Various surgical techniques have been used at different institutions. Minimally invasive techniques and laparoscopic procedures are being increasingly reported [13, 14]. Typically, perforations are temporarily closed. However, in cases of severe surrounding infection, a T-tube or other drainage method may be necessary [15, 16]. When vascularity at the perforation margins is poor or severe infection is present, reinforcement with an omental patch has been reported to be effective [17]. Esophagectomy is required in cases where primary closure is not feasible or when extensive necrosis of the esophagus is present [18].

In the present case, the patient presented in a critical condition with shock-like vital signs. Additionally, the presence of thoracic perforation alongside esophageal varices necessitated not only infection control but also bleeding suppression. Therefore, surgery was the chosen course of treatment. Previous reports on the surgical management of Boerhaave syndrome have often employed primary closure of the perforation site. However, studies have shown a high rate of suture failure in cases involving intrathoracic perforation, particularly in those caused by contamination [1]. In our case, we opted for T-tube insertion because of concerns regarding severe infection. The large number of blood clots from ruptured esophageal varices, combined with potential exposure to gastrointestinal contents from the perforation, created a high-risk environment for infection. The perforation site was sufficiently reinforced with nearby pleural and pericardial fat; therefore, an omental patch was not required. Although T-tube insertion prolonged the hospital stay, the patient was discharged without any postoperative complications. Our case is unique, as it presented with both Boerhaave syndrome and esophageal variceal rupture—conditions that have not been reported together before.

We propose the following approach for cases in which Boerhaave syndrome complicated with ruptured esophageal varices. When active variceal bleeding is present, bleeding control should take precedence. If a bleeding source is identified at operation, it should be controlled by ligation or oversewing. After hemostasis has been secured, definitive management can proceed according to standard practice for isolated Boerhaave syndrome, based on the degree of contamination and the condition of the tissues.

In this case, endoscopic hemostasis was attempted but unsuccessful, so we proceeded to surgery. Intraoperatively, no discrete bleeding source was identified; therefore, hemostasis was achieved by placing broad single-layer sutures incorporating the pleura and the margins of the esophageal perforation.

A previous report described Boerhaave syndrome complicated by liver cirrhosis and esophageal varices. In one case involving a 28-year-old man, conservative management was initially chosen; however, 7 days after presentation, the patient underwent thoracic esophagectomy and esophagostomy. Subsequently, reconstruction with a gastric conduit was performed using a second-stage procedure [19]. There is a report of favorable outcomes with conservative management in cases of esophageal perforation following Sengstaken–Blakemore (SB) tube placement for ruptured esophageal varices. However, in this case, the perforation was relatively small [20].

This case suggests that preexisting esophageal varices may increase the risk of esophageal rupture. Sclerotherapy, a common treatment for varices, may also contribute to esophageal wall weakness, although the evidence is limited. Notably, the perforation was located at the 2 o’clock position and appeared to coincide with the site of a previous EVL ligation.

Our comprehensive search revealed no previous reports of Boerhaave syndrome co-occurring with esophageal variceal rupture in patients with liver cirrhosis. This unique case presentation raises the question of whether esophageal varices predispose patients to spontaneous rupture.

There are two potential explanations for this finding. First, the presence of varices weakens the esophageal wall. Second, previous treatments for varices, such as sclerotherapy, may have contributed to the esophageal wall weakness. While the evidence is limited, Clause et al*.* reported a case of esophageal perforation following sclerotherapy for varices, which was successfully treated with stent placement and perforation closure [21].

Boerhaave syndrome can occur with vomiting after heavy drinking, and esophageal varices have been found to complicate alcoholic cirrhosis. Consequently, cases of simultaneous varices and esophageal rupture are likely to continue. Patients with varices may be more prone to esophageal rupture due to vomiting after heavy alcohol consumption; therefore, care should be taken regarding the amount of alcohol consumed.

In conclusion, Boerhaave syndrome, a life-threatening esophageal rupture, can occur due to forceful vomiting and is often associated with heavy alcohol consumption. We report a case of Boerhaave syndrome with hemorrhage, in which the patient underwent surgery and had a favorable outcome. Patients with esophageal varices should be cautious about alcohol consumption to minimize the risk of Boerhaave syndrome.

## Data Availability

The datasets generated during the current study are not publicly available due to institutional privacy regulations and ethical restrictions protecting patient‐identifiable clinical information, but de-identified data may be obtained from the corresponding author on reasonable request and with approval of the Institutional Review Board of Gunma University Hospital.

## Abbreviation

CT: Computed tomography
